# Allysine and α-Aminoadipic Acid as Markers of the Glyco-Oxidative Damage to Human Serum Albumin under Pathological Glucose Concentrations

**DOI:** 10.3390/antiox10030474

**Published:** 2021-03-17

**Authors:** Carolina Luna, Alexis Arjona, Carmen Dueñas, Mario Estevez

**Affiliations:** 1 Emergency unit, Hospital Nuestra Señora de la Montaña, Servicio Extremeño de Salud, Gobierno de Extremadura, 10002 Cáceres, Spain; clunaest@alumnos.unex.es; 2 Family and Community Medicine, Servicio Extremeño de Salud, Gobierno de Extremadura, 10002 Cáceres, Spain; alexis.arjona@salud-juntaex.es; 3 Gastroenterology unit, Hospital Universitario Cáceres, Servicio Extremeño de Salud, Gobierno de Extremadura, 10002 Cáceres, Spain; carmen.duenas@salud-juntaex.es; 4 Meat and Meat Products Research Institute (IPROCAR), Food Technology, University of Extremadura, 10003 Cáceres, Spain

**Keywords:** allysine, α-aminoadipic acid, AGEs, diabetes, protein oxidation, Maillard reaction

## Abstract

Understanding the molecular basis of the disease is of the utmost scientific interest as it contributes to the development of targeted strategies of prevention, diagnosis, and therapy. Protein carbonylation is a typical feature of glyco-oxidative stress and takes place in health disorders such as diabetes. Allysine as well as its oxidation product, the α-amino adipic acid (α-AA) have been found to be markers of diabetes risk whereas little is known about the chemistry involved in its formation under hyperglycemic conditions. To provide insight into this issue, human serum albumin was incubated in the presence of FeCl3 (25 μM) and increasing glucose concentrations for 32 h at 37 °C. These concentrations were selected to simulate (i) physiological fasting plasma concentration (4 mM), (ii) pathological pre-diabetes fasting plasma concentration (8 mM), and pathological diabetes fasting plasma concentration (12 mM) of glucose. While both allysine and α-AA were found to increase with increasing glucose concentrations, the carboxylic acid was only detected at pathological glucose concentrations and appeared to be a more reliable indicator of glyco-oxidative stress. The underlying chemical mechanisms of lysine glycation as well as of the depletion of tryptophan and formation of fluorescent and colored advanced glycation products are discussed.

## 1. Introduction

The oxidative damage to proteins is an expression of the impairment of the redox status and typically takes place during oxidative stress, aging, and the onset of age-related diseases [1]. The accretion of oxidized proteins in tissues as a result of enduring oxidative stress is considered to contribute to cell dysfunction and health disorders [2]. Carbonylation is commonly regarded as one of the most remarkable expressions of protein oxidation [3]. The formation of protein carbonyls is usually described as the result of a radical-mediated mechanism by which sensible alkaline amino acids (lysine, proline, arginine) undergo oxidative deamination to yield aldehydes, namely, the α-aminoadipic semialdehyde (also known as allysine) and the γ-glutamic semialdehyde [3]. However, the reaction of reducing sugars and Maillard-derived dicarbonyls (i.e., glyoxal and methyl-glyoxal) with such alkaline amino acids have also been found to lead to the formation of the aforementioned aldehydes [4]. In fact, this Maillard pathway of protein carbonylation has recently been emphasized as remarkable compared to the radical-mediated mechanisms in conditions that simulate both physiological and food processing [5,6]. The molecular nature of oxidative and glyco-oxidative reactions in proteins has been poorly studied even though their occurrence and biological consequences are known to be highly relevant. Uncontrolled levels of circulating glucose and glyoxal are responsible for the onset of cardiometabolic conditions via a plausible induction of glyco-oxidative stress and massive protein damage [7].

In recent years, an intermediate compound in lysine metabolism, the α-aminoadipic acid (α-AA), has been surprisingly identified as a biomarker of insulin resistance and obesity [8]. Earlier, this compound was identified as an early indicator of diabetes in a metabolomic study in humans and was found to induce such metabolic conditions in experimental animals after oral administration [9]. These authors identified α-AA as a “poorly characterized degradation product of lysine” and stated that previous authors included this species as part of a carbonyl stress pathway in diabetes [10,11]. A recent study has reported the ability of α-AA to impair the redox status and functionality of intestinal and pancreatic cells, confirming the implication and potential toxicity of this metabolite in the pancreas and glucose regulation [12,13]. Yet, the molecular basis of the implication of this metabolite in the onset of type II diabetes is poorly understood. Interestingly, α-AA is the final oxidation product from allysine and its formation in human and food proteins has been confirmed in simulated radical-generating systems [14,15]. The formation of its precursor in the presence of glucose and toxic diabetes metabolites (glyoxal) suggests that α-AA may be formed under pathological pro-diabetic conditions, drawing the link between this species and the metabolic disorder [16]. Yet, no previous study has investigated the molecular mechanisms behind the formation of α-AA through the Maillard-pathway.

This study was designed to investigate the formation of allysine and α-AA in human serum albumin in the presence of increasing concentrations of glucose and assess the suitability of these species as reliable markers of the glyco-oxidative damage to human plasma proteins.

## 2. Material and Methods

### 2.1. Chemicals, Reagents, and Materials

All chemicals were supplied from Panreac (Panreac Quimica, S.A., Barcelona, Spain), Merck (Merck, Darmstadt, Germany), and Scharlau (Gillman, South Australia). In particular, human serum albumin (CAS n° 70024-90-7), α-AA (CAS n° 542-32-5), and allysine precursor (Nα-acetyl-l-lysine; CAS n° 1946-82-3) were purchased from Sigma Chemicals (Merck, Darmstadt, Germany). The water used in the experiments was purified by passage through a Milli-Q system (Millipore Corp., Bedford, MA, USA). Allysine standard was synthesized in the laboratory following the procedure described by Akagawa et al. [4]. The concentration of all reactants are referring to the final concentration in the reaction mixture.

### 2.2. Experimental Design

Human serum albumin (HSA; 5 mg/mL), was dissolved in 100 mM phosphate buffer (pH 6.5) and subsequently incubated with FeCl_3_ (25 μM) and increasing glucose concentrations. These concentrations were selected to simulate i) physiological fasting plasma concentration (4 mM), ii) pathological pre-diabetes fasting plasma concentration (8 mM), and pathological diabetes fasting plasma concentration (12 mM) of the monosaccharide. Fe^3+^ was added to promote glucose oxidative degradation. Reaction mixtures were allowed to incubate at 37 °C in an oven at constant stirring for 32 h. At fixed times (0, 4, 8, 24, 28, and 32 h) the experimental units were sampled and analyzed for glucose consumption. At intermediate sampling times (4, 8, 24, and 28 h), the depleted glucose concentration was restored by immediately adding additional glucose to guarantee that all experimental units had their corresponding initial glucose concentrations (4, 8, and 12 mM). In addition to glucose concentration, experimental units were analyzed at all sampling times for tryptophan depletion, and formation of allysine, α-AA, advanced glycation end-products (AGEs), and yellowness. The entire experiment was replicated three times (true replicates) in corresponding independent assays and all analyses were repeated three times (technical replicates) in each experimental unit, totaling 9 measurements per analysis, treatment, and sampling time. These data were used to calculate means and standard deviations and subjected to statistical analysis.

### 2.3. Analysis of Samples

#### 2.3.1. Glucose Analysis by Spectrophotometry

Glucose was quantified using the 3,5-dinitrosalicylic acid (DNS) in accordance with the method reported by Miller [17] with minor modifications as follows. Two hundred microliters of sample were centrifuged for 3 min at 805 *g* using polypropylene tubes with nylon filters (0.45 μm pore size, Merck KGaA, Darmstadt, Germany). Filtrates were brought to volume in 25 mL volumetric flasks. Five hundred microliters were added to 0.5 mL of 1% DNS and placed in a boiling water bath (100 °C) for 5 min. After cooling, 5 mL of distilled water were added to the reaction mixture, and absorbance was measured at 540 nm. Glucose was quantified at each sampling point using a standard curve of glucose analyzed under the same conditions (0–1 mg/mL). To calculate the glucose consumed in the reaction, glucose concentration (quantified) at each sampling was subtracted to the initial glucose concentrations of each experimental unit. Results were expressed as mM of glucose consumed at each sampling point.

#### 2.3.2. Analysis of Allysine by HPLC

Two hundred microliters of sample were treated with 1 mL 10% TCA in 2 mL Eppendorf microtubes. Pellets were collected by centrifugation at 2000 *g* for 30 min at 4 °C and treated again with 1 mL 5% TCA. Upon centrifugation (5000 *g* for 5 min at 4 °C), pellets were treated with freshly prepared (i) 0.5 mL 250 mM 2-(N-morpholino) ethanesulfonic acid (MES) buffer pH 6.0 with 1% sodium dodecyl sulfate (SDS) and 1 mM diethylenetriaminepentaacetic acid (DTPA); (ii) 0.5 mL 50 mM ABA in 250 mM MES buffer pH 6.0; (iii) 0.25 mL 100 mM NaBH_3_CN in 250 mM MES buffer pH 6.0. Derivatization was allowed to occur for 90 min at 37 °C and the samples were stirred every 15 min. Upon completion, pellets were collected upon treatment with 1 mL 50% TCA solution and centrifuged at 5000 *g* for 10 min. Pellets were then washed twice with 1.5 mL 10% TCA and diethyl ether-ethanol (1:1). Finally, proteins were hydrolyzed in the presence of 6 N HCl at 110 °C for 18 h. The hydrolysates were dried in a centrifugal evaporator in vacuo. The hydrolysates were dissolved in 200 μL Milli-Q water and then filtered through hydrophilic polypropylene GH Polypro (GHP) syringe filters (0.45 μm pore size, Pall Corporation, NJ, USA) for HPLC analysis.

A Shimadzu ‘Prominence’ HPLC apparatus (Shimadzu Corporation, Kyoto, Japan), equipped with a quaternary solvent delivery system (LC-20AD), a DGU-20AS on-line degasser, a SIL-20A auto-sampler, an RF-10A XL fluorescence detector (FLD), and a CBM-20A system controller, was used. An aliquot (1 μL) from the reconstituted protein hydrolysates was injected and analyzed in the above-mentioned HPLC equipment. AAS-ABA and GGS-ABA were eluted in a Cosmosil 5C18-AR-II RP-HPLC column (5 µm, 150 × 4.6 mm) equipped with a guard column (10 × 4.6 mm) packed with the same material. The flow rate was kept at 1 mL/min and the temperature of the column was maintained constant at 30 °C. The eluate was monitored with excitation and emission wavelengths set at 283 and 350 nm, respectively. Allysine standard (0.1 μL) was run and analyzed under the same conditions and its retention time compared with that from samples for identification. The peak corresponding to allysine was integrated and the resulting areas plotted against an ABA standard curve (0.1 to 0.5 mM) as proposed by Utrera et al. [18]. Results are expressed as nmol of allysine per mg of protein.

#### 2.3.3. Analysis of α-AA by HPLC

One hundred microliters of sample were treated with 1 mL 10% TCA in 2mL Eppendorf microtubes. Pellets were collected by centrifugation at 2000 *g* for 30 min at 4 °C and subsequently hydrolyzed with 3 N HCl and 110 °C. Hydrolysates were derivatized by 0.2 mM 9-fluorenylmethyl chloroformate (FMoc) according to the procedure described by Utrera and Estévez [19]. The derivatized samples were filtered through hydrophilic polypropylene GH Polypro (GHP) syringe filters (0.45 μm pore size, Pall Corp., New York, USA) for HPLC analysis. An aliquot (1 μL) was injected and analyzed in the above-mentioned HPLC equipment (Shimadzu Corp., Kyoto, Japan) using a Zorbax Eclipse AAA column (Agilent, CA, USA) (3.5 μm, 4.6 mm × 150 mm) and a guard column (10 × 4.6 mm) filled with the same material. Eluent A was 20 mM ammonium acetate (pH 6.5) and 15% methanol, and eluent B was 90% acetonitrile. The flow rate was constant at 1.0 mL/min, and the column was maintained at 35 °C. The gradient profile was as follows: 0–1.5 min, 12% B; 1.5–2.0 min, 12–18% B; 2.0–9.0 min, 18% B; 9.0–9.5 min, 18–25% B; 9.5–12.5 min, 25% B; 12.5–13.0 min, 25–30% B; 13.0–16.0 min, 30% B; 16.0–17.0 min, 30–40% B; 17.0–20.0 min, 40% B; 20.0–22.0 min, 40–50% B; 22.0–23.0 min, 50% B; 23.0–24.0 min, 50–99% B. Excitation and emission wavelengths were set at 263 and 313 nm, respectively. Identification of the derivatized α-AA in the FLD chromatograms was carried out by comparing its retention time with that from a standard compound injected and analyzed in the above-mentioned conditions. The peaks corresponding to α-AA–FMoc were integrated and the resulting areas were plotted against an α-AA–FMoc standard curve (0.4 to 5 pM). Results were expressed as nmol of α-AA per g of protein. FLD chromatograms of FMOC-α-AA standard compound (A) and of a real sample in which FMOC-α-AA is manually integrated (B) are available in Figure S1 as supplementary material.

#### 2.3.4. Analysis of Tryptophan by Fluorescence Spectroscopy

Tryptophan depletion in HSA was analyzed by fluorescence spectroscopy following the procedure described by Arcanjo et al. [16]. Samples were diluted (1:1000; v:v) with a 0.05 M Tris-HCl buffer pH 7.5. Diluted samples were dispensed in quartz cuvettes and excited at 283 nm on a Perkin Elmer LS 55 luminescence spectrometer (PerkinElmer, Beaconsfield, UK). The emission spectra were recorded from 300 to 400 nm Emission spectra of the Tris-HCl buffer were recorded under the same conditions and used as background spectra. Excitation and emission slit widths were set at 10 nm and data was collected at 500 nm per minute. Tryptophan content was calculated from a standard curve of N-acetyl-l-tryptophanamide (NATA; 0.1 to 0.5 μM). Results are expressed as μmol of tryptophan per L of suspension.

#### 2.3.5. Analysis of AGEs by Fluorescence Spectroscopy

AGEs were analyzed using the fluorescence spectrometer aforementioned (Perkin-Elmer, Beaconsfield, UK). Prior to the analysis, HSA suspensions were diluted with 100 mM sodium phosphate buffer, pH 7.4. AGEs were excited at 350 nm, and the emitted fluorescence was recorded from 400 to 500 nm. The excitation and emission slits were both set to 10 nm and the scanning speed was 500 nm/min. Results are expressed as fluorescence intensity (Area units).

#### 2.3.6. Analysis of Yellowness by Spectrophotometry

The formation of colored pigments as a result of the onset of the glycation of HSA was determined by using the weighted-ordinate method by Hunter [20] as modified by Luna and Estévez [5]. Transmittances from 400 to 700 nm at constants intervals of 10 nm were measured in 1 mL of protein suspensions by spectrophotometry. Data were used to calculate tristimulus values (X, Y, Z) and subsequently transformed into the CIELAB L *, a *, and b * color values. The yellowness index was calculated at sampling times using the following formula: yellowness index = 142.86b */L *.

#### 2.3.7. Statistical Analysis

Data from the analysis (3 true replicates × 3 technical replicates = 9 data per treatment and sampling point) were collected and subjected to statistical analyses. Data was found to be normal (Shapiro–Wilk test) and homoscedastic (Bartlett’s test). To assess the effect of the increased glucose concentration on the measured parameters, an analysis of variance (ANOVA) was applied to data within each sampling time. To assess the effect of sampling time on measured parameters, repeated-measures ANOVA was applied. Tukey tests were applied when ANOVAs found significant differences between groups of samples. The statistical significance was set at *p* < 0.05 (SPSS v. 15.5).

## 3. Results and Discussion

### 3.1. Carbonylation of HSA by Glucose

**Figure 1:**
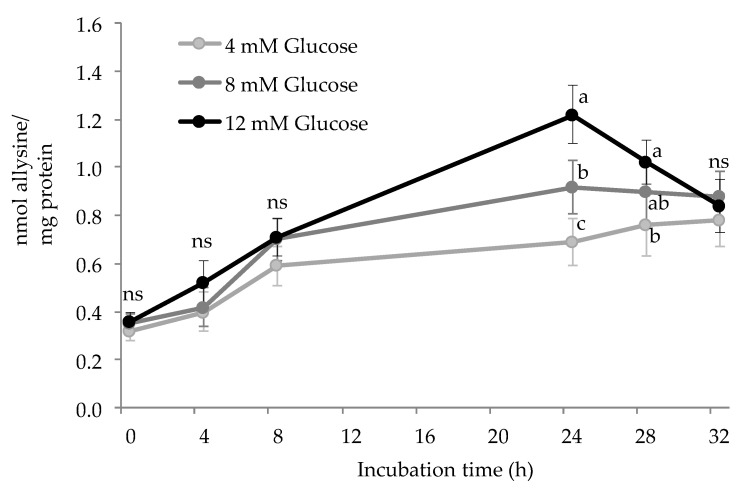
Protein carbonylation (nmol allysine/mg protein) in HSA solution (5 mg/mL; 37 °C; 32 h) incubated with increasing concentrations of glucose. Different letters (a–c) at the same sampling time denote significant differences (*p* < 0.05) between treatments. Ns: no significant differences.

Figure 1 shows the accretion of allysine in HSA during incubation with increasing glucose concentrations (4, 8, and 12 mM) and 25 μM of Fe^3+^. Carbonylation of HSA took place at all glucose concentrations, which confirms the oxidative degradation mechanism of lysine residues by the Maillard pathway as originally reported by Akagawa et al. [21]. These authors reported the carbonylation of bovine serum albumin (BSA) suspensions incubated with 50 mM glucose and 5 μM Cu^2+^ at 37 °C for 3 weeks. It is worth noting that the aforementioned study applied a more severe and less realistic pro-oxidative environment than those from the present study. Using the same glucose concentration, Luna and Estévez [6] were able to induce the formation of allysine in HSA (37 °C/24 h). These authors reported that this glycosylation mechanism was as efficient in causing carbonylation in HSA as a hydroxyl-radical generating system (0.6 mM H_2_O_2_/0.1 mM Fe^3+^).

**Figure 2:**
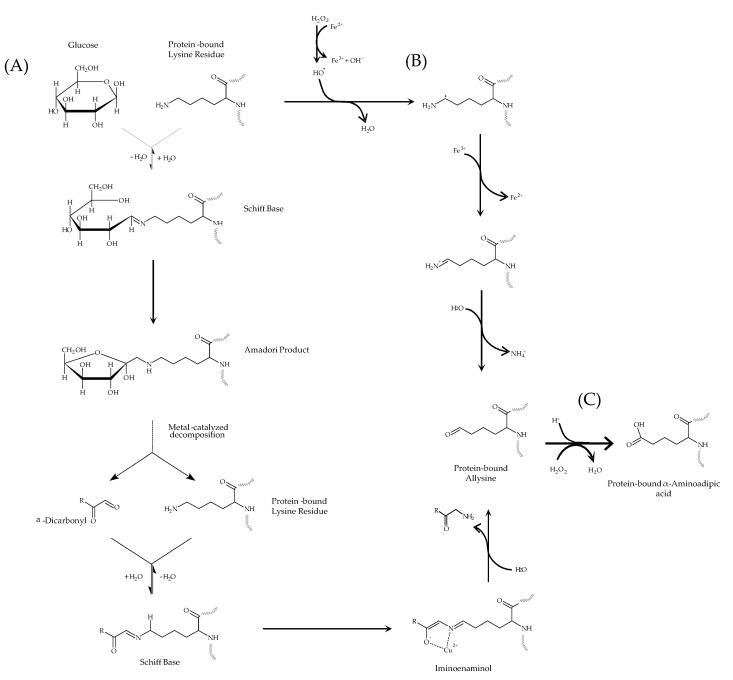
(**A**) Carbonylation of a protein-bound lysine residue in the presence of glucose and transition metals in accordance with the mechanisms proposed by Suyama et al. [22]. (**B**) Carbonylation of a protein-bound lysine residue in the presence of a hydroxyl-radical generating system as proposed by Akagawa and Suyama [23]. (**C**) Formation of α-AA from a protein-bound allysine residue in the presence of peroxide as proposed by Utrera and Estévez [14].

As depicted in Figure 2, both carbonylation mechanisms lead to the formation of the same oxidation product (allysine) which signifies the interconnection between radical-mediated oxidative damage to proteins and the Maillard reaction. In the latter pathway (Suyama pathway, A), a protein-bound lysine residue reacts with glucose to yield, in the presence of transitions metals such as iron, α-dicarbonyl compounds. Dicarbonyls attack the ε-amino group from lysine residues to form a Schiff base adduct (an iminoketone). An iminoenaminol is subsequently formed to eventually hydrolyze to yield an enaminol and allysine.

This Maillard-mediated mechanism of carbonylation has been replicated by authors by using Maillard α-dicarbonyl such as glyoxal (GO) and methylglyoxal (MGO) as reactants, proving that these species are active promoters of allysine formation in proteins. Suyama et al. [22] studied BSA carbonylation in the presence of 50–200 mM GO and MGO and 500 μM Cu^2+^ (37 °C/14 days) and Luna and Estévez [5] showed the effect of 2 M of GO/MGO in β-lactoglobulin and myofibrillar proteins (80 °C/48 h). In line with the present experiment, Arcanjo et al. [16] reported the oxidative damage caused to HSA by biologically relevant concentrations of GO and MGO (0.4 mM/37 °C/48 h) proving that this mechanism may occur in vivo, explaining the increased carbonylation levels in plasma proteins from animals and humans with impaired glucose metabolism. It is worth highlighting that the radical-mediated pathway of protein carbonylation (represented in Figure 2 as pathway B) cannot be ruled out since the oxidative decomposition of glucose in the presence of transition metals such as iron leads to the formation of a variety of reactive oxygen species (ROS) and hydrogen peroxide [24]. The role of Fe^3+^ in such degradation is well-known and involves the acceptance of the electrons lost by glucose and Maillard intermediates such as Amadori products. In a recent study [25] ROS was found to be formed during simulated digestion of proteins in the presence of glucose and heme iron. As reported by Luna and Estévez [6], if both carbonylation pathways are taking place at the same time and it leads to the formation of the same oxidation product (allysine), the contribution of each of them to protein carbonylation is indistinguishable.

**Figure 3:**
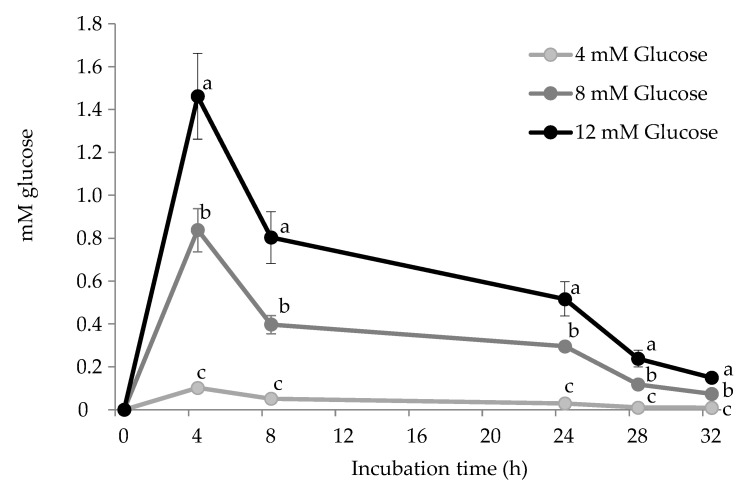
Glucose (mM) consumed during incubation of HSA solutions (5 mg/mL; 37 °C; 32 h) with increasing concentrations of the glucose. To calculate the glucose consumed in the reaction at each sampling point, the remaining glucose (quantified) was subtracted to the initial glucose concentrations of each experimental unit. Different letters (a–c) at the same sampling time denote significant differences (*p* < 0.05) between treatments.

In the present experiment, allysine concentrations increased significantly in all experimental units (*p* < 0.05) while the evolution greatly depended on the glucose concentration. Whereas no differences between groups were observed during the first 8 h, the concentration of allysine peaked at 24 h, where a clear and significant dose-effect of glucose of allysine formation was observed (*p* < 0.05). At 24 h, allysine concentration in HSA challenged with pathological glucose levels (12 mM) almost doubled (1.22 nmol/mg protein) that found in HSA exposed to physiological ones (4 mM) (0.68 nmol/mg protein). After this sampling point, allysine concentration seems to find stability in HSA incubated with 4 and 8 mM glucose while a significant decrease was observed in HSA exposed to 12 mM. Allysine decline in HSA exposed to 12 mM glucose may respond to its involvement in further reactions including a further oxidation step to yield α-AA (pathway C in Figure 2) and the formation of AGEs, among others [3,4]. Further details of allysine reactivity are provided in due course. At the end of the assay, similar allysine concentrations (~0.8 nmol/mg protein) were found in all experimental units. The evolution of allysine in experimental units is consistent with glucose consumption shown in Figure 3.

Surprisingly, only a small share of the added glucose was consumed in the reaction, suggesting that its implication was likely limited by its reactivity with Fe^3+^ and with the protein-bound amino residues. It is worth noting that subsequent glucose additions to restore initial concentrations did not affect the consumption of this reactant that dropped after each sampling. Yet, the percentage of consumed glucose at 4 h was in line with glucose concentration, with this being 12%, 10%, and 2.5% in experimental units added with 12 mM, 8 mM, and 4 mM glucose, respectively. Also using biologically relevant glucose concentrations (12 mM, 0.2 mM Fe^3+^), Ozyurt et al. [26] already observed the extent to which hyperglycemic conditions lead to the formation of allysine in human plasma proteins (37 °C/10 days). These scientific pieces of evidence explain the molecular mechanisms behind the carbonylation of plasma proteins that are known to occur in patients with sustained hyperglycemia [27]. While allysine may be a marker of the glyco-oxidative damage to proteins in type II diabetes patients, the oxidative stress caused by elevated glucose circulating levels may trigger the molecular mechanisms behind the onset of the metabolic syndrome [28]. Post-translational changes in proteins, and carbonylation, in particular, affect protein conformation and biological function [28]. The extent of protein carbonylation, greatly influenced by reacting glucose, as observed in the present study, affects homeostasis and the control of metabolic processes [28]. The understanding of the molecular basis of protein carbonylation and its role in type II diabetes may be relevant for antiglycation and antioxidant therapeutic options. Yet, the reactivity of allysine, reflected in the present experiment as the depletion of such carbonyl at the end of the assay, suggests that other advanced products of lysine glyco-oxidation may be more reliable indicators of pathological conditions caused by impaired glucose metabolism.

### 3.2. Formation of α-AA in HSA by Glucose

**Figure 4:**
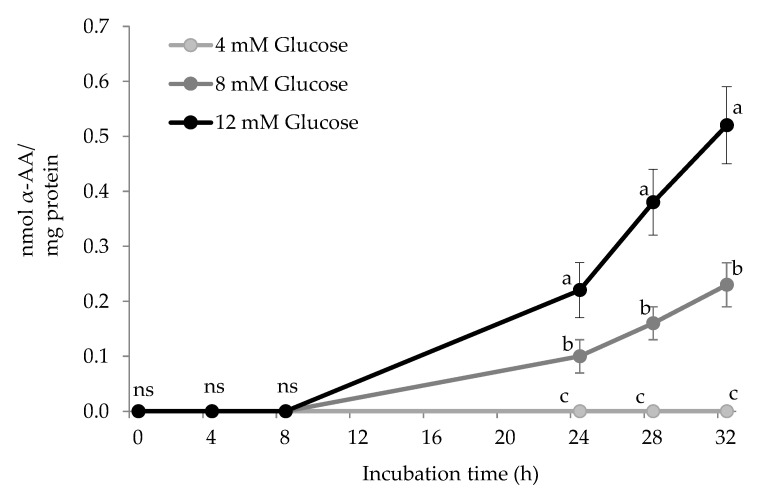
α-Aminoadipic acid (α-AA) concentration (nmol α-AA/mg protein) in HSA solution (5 mg/mL; 37 °C; 32 h) incubated with increasing concentrations of glucose. Different letters (a–c) at the same sampling time denote significant differences (*p* < 0.05) between treatments. Ns: no significant differences.

Figure 4 shows the accretion of α-AA in HSA during incubation with increasing glucose concentrations (4 mM, 8 mM, and 12 mM) and 25 μM of Fe^3+^. No α-AA was detected in HSA solutions during the first three sampling points. From 24 h onwards, the concentration of α-AA progressively increased in HSA exposed to 8 mM and 12 mM glucose. The concentration of α-AA in HSA exposed to the physiological glucose levels remained below the detection limit during the entire assay. Significant differences were found between groups, with these showing a clear dose-effect of glucose concentration on α-AA accretion. α-AA is known to be a final and more stable oxidation-end product of lysine. As shown in Figure 2C, the formation of α-AA from its precursor, allysine, requires the participation of peroxides such as hydrogen peroxide or organic peroxides such as those formed from lipid and protein peroxidation. These species are present in biological samples subjected to pro-oxidative conditions and are expected to occur in the plasma of patients with elevated glucose levels and hence, suffering from oxidative stress and metabolic syndrome [2,28]. In the conditions of the present experiment, hydrogen peroxide may have been produced as a result of the reactivity of iron with O_2_ and other reactive and non-reactive oxygen species. Furthermore, as previously stated, Wang et al. [24] and subsequently ourselves [25] reported the formation of a variety of ROS as a result of the oxidative decomposition of reducing sugars in the presence of transition metals such as iron.

The pro-oxidative environment could have caused the formation of protein peroxides that could also be involved in the transition of allysine to α-AA. The timely temporal and quantitative coincidence during the assay between allysine depletion and α-AA formation in experimental units exposed to 12 mM glucose provides strength to the hypothesis that α-AA formation took place according to the aforementioned mechanisms and, specifically from allysine oxidation (pathway C in Figure 2). The in vitro formation of α-AA by exposure to hydroxyl-radical generating systems has already been documented in proteins from plasma and other tissues [10] and in food proteins [14,15]. α-AA formation has also been reported to occur in vivo via myeloperoxidase-catalyzed oxidation of lysyl residues in HSA and other tissue proteins [29]. Nevertheless, this is, to our knowledge, the first time that α-AA is reported to be formed in HSA under simulated hyperglycemic conditions. α-AA was already proposed as a reliable marker of oxidative stress, aging, and disease [10]. As stated by Sell et al. [10], α-AA is, unlike its precursor, allysine, a stable final product from lysine, and this is actually corroborated by our present results. Recent studies emphasized the role of α-AA as an early indicator of type 2 diabetes [8,30]. The results from the present study underline the molecular connection between elevated glucose concentrations and the formation of α-AA in HSA. Supporting the idea of α-AA being a more reliable indicator of allysine of the glyco-oxidative damage caused by circulating glucose to HSA, only pathological glucose concentrations (>7 mM) were able to yield measurable amounts of α-AA. Moreover, α-AA accretion was found to be stable over time and dependent on glucose concentration, which emphasizes its use as a consistent biological marker. Despite the large differences between the current in vitro conditions and those that occurred in vivo, the concentration of α-AA reported in the present study are consistent with those found in plasma from obese patients under risk of insulin resistance [8].

### 3.3. Tryptophan Depletion by Glucose

**Figure 5:**
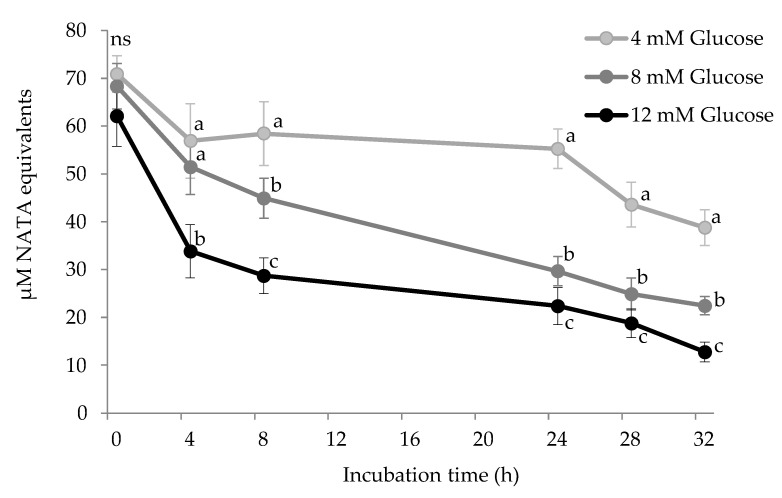
Tryptophan concentration (µM) in HSA solution (5 mg/mL; 37 °C; 32 h) incubated with increasing concentrations of glucose. Different letters (a–c) at the same sampling time denote significant differences (*p* < 0.05) between treatments. Ns: no significant differences.

Tryptophan is known to be particularly susceptible to pro-oxidative environments and its degradation is known to occur as a primary manifestation of the oxidative damage to proteins [1]. The depletion of tryptophan in HSA during incubation with increasing glucose concentrations (4 mM, 8 mM, and 12 mM) and 25 μM of Fe^3+^, is shown in Figure 5.

The initial tryptophan concentrations declined during the assay with the percent loss being 20.6%, 32.8% and 54.7% in HSA incubated with 4 mM, 8 mM, and 12 mM glucose, respectively. The depletion of protein-bound tryptophan-induced by a glyco-oxidative system has been already documented. However, some of these previous in vitro assays differ from the present one for inducing more severe and less realistic physiological conditions [31] or for using food proteins [6]. For instance, Khan et al. [31] recorded tryptophan depletion in glycated HSA for up to 20 weeks in the absence of metals. Yet, transition metals such as iron or copper catalyze glucose oxidation and may promote, as a result, tryptophan depletion. As aforementioned, glucose undergoes metal-catalyzed oxidation that leads to the production of ROS such as hydrogen peroxide, dicarbonyls, and other highly reactive pro-oxidants [24]. Consistently, Coussons et al. [32] confirmed the remarkable effect of Cu^2+^ (50 μM) on tryptophan depletion in HSA exposed to glucose (5–50 mM). In blood, iron may play a similar role and heme plasma proteins such as hemoglobin have been reported to be particularly susceptible to undergo oxidative damage. Cussimanio et al. [33] and Özyurt et al. [26] reported the high susceptibility of tryptophan residues from hemoglobin to be readily oxidized by glucose-mediated ROS. In line with the present results, Özyurt et al. [26] also reported tryptophan depletion in HSA during incubation at simulated hyperglycemic conditions (12 mM glucose/0.2 mM Fe^3+^). As well as carbonylation of plasma proteins, the loss of tryptophan in plasma proteins from individuals with impaired glucose metabolism is an event of biological significance. Scientific evidence indicates decreased tryptophan levels in the plasma of diabetic patients [34] and the molecular basis of this depletion may be those exposed in the present study. Given the numerous and relevant biological functions of tryptophan and its metabolites, this depletion may cause further physiological disorders such as coronary diseases [35] and neurological complications [36]. On the same line, Rebnord et al. [35] proposed using the kynurenine:tryptophan ratio as a predictor of early type 2 diabetes. The understanding of the molecular mechanisms underlying the loss of tryptophan in patients with hyperglycemia may assist in searching for targeted diagnostic and therapeutic tools.

### 3.4. Other Expressions of Protein Glycoxidation: AGEs and Yellowness

**Figure 6:**
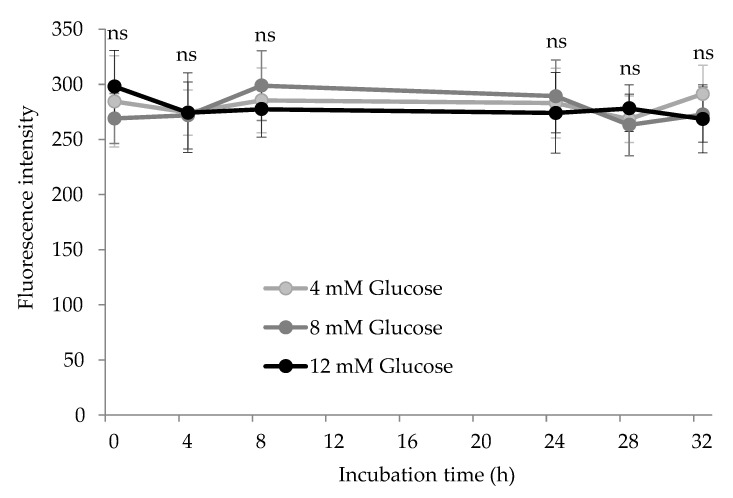
Advanced glycation end-product (AGE) concentration (fluorescent units) in HSA solution (5 mg/mL; 37 °C; 32 h) incubated with increasing concentrations of glucose. Ns: no significant differences between treatments at the same sampling time.

**Figure 7:**
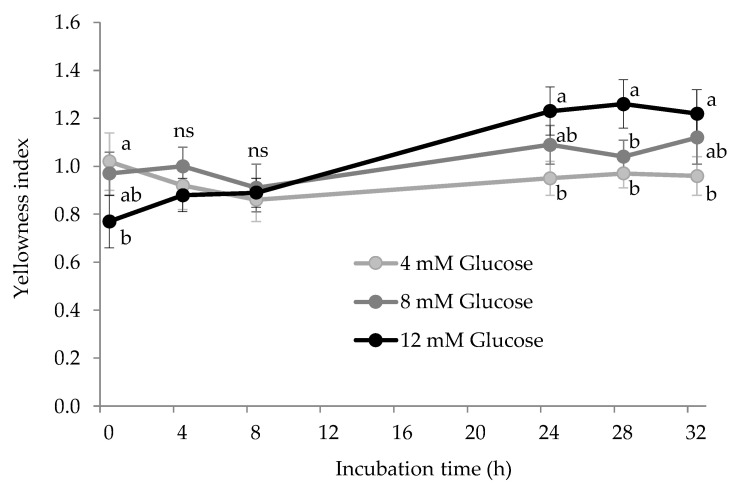
Yellowness (dimensionless) in HSA solution (5 mg/mL; 37 °C; 32 h) incubated with increasing concentrations of glucose. Different letters (a–c) at the same sampling time denote significant differences (*p* < 0.05) between treatments. Ns: no significant differences.

Finally, the glycation system also induced the accretion of AGEs (Figure 6) and other fluorescent and colored pigments in HSA, spectrophotometrically measured as yellowness (Figure 7). AGEs are recognized as a remarkable expression of glycosative stress on plasma proteins [37].

Unlike the aforementioned markers of glyco-oxidation, AGEs are formed via condensation of various chemical species by assorted pathways. Despite being a heterogeneous group of macromolecules generated at advanced stages of the Maillard reaction, their biological effects are remarkable. They have been found to induce cellular toxicity and contribute to complications in diabetic patients [37]. Their formation under physiological conditions takes several months of sustained exposure to circulating glucose while it is accelerated in hyperglycemia conditions [38]. This may be the reason why we observed significant changes in the concentration of AGEs during the assay. No significant differences were neither found between treatments, as glucose concentration had no impact on this measurement. In a previous study in which blood proteins were exposed to 12 mM glucose for 10 days at 37 °C, AGEs were found to increase in both HSA and hemoglobin [26]. In addition to the longer duration of the aforementioned assay, the authors added 8-times larger concentrations of Fe^3+^ (200 μM) which could have contributed to creating a more severe pro-oxidative environment and hence, a more intense formation of AGEs. While fluorescent AGEs did not seem to be affected by glucose exposure, the formation of advanced colored products took place as measured by the yellowness index. As shown in Figure 7, the yellowness index significantly increased over time in experimental units with the highest glucose concentration (12 mM) and remained unchanged in samples with lower glucose concentrations. Significant differences between suspensions with 4 mM and 12 mM were found at the last three sampling points. The formation of yellow-brown products is a typical feature of the Maillard reaction and commonly used as an indicator of non-enzymatic browning in food systems [5,39]. Yet, this is rarely employed to assess glyco-oxidative stress in other biological samples such as a plasma or other tissues. These results emphasize that AGEs may not be as specific and reliable markers of glyco-oxidative stress as lysine oxidation products and yellowness measurement is found as an inexpensive and fast procedure for a qualitative evaluation of the extent of glycation of HSA.

## 4. Conclusions

The exposure of HSA to simulated hyperglycemic conditions leads to assorted chemical modifications with carbonylation, the formation of α-AA, and tryptophan depletion being some of the most remarkable. α-AA is originally reported to be formed in human plasma proteins in the presence of pathological glucose concentrations and the likely chemical pathways and mechanisms are discussed. α-AA is, unlike its precursor allysine, a stable chemical species and could be considered a reliable indicator of diabetes and other health disorders associated with impaired glucose metabolism and hyperglycemia. Further studies are required to investigate the biological consequences of these protein modifications as they may play a role in the onset of the physiological impairments that occurred in patients with diabetes.

## Data Availability

Not applicable.

## Supplementary Materials

The following are available online at https://www.mdpi.com/2076-3921/10/3/474/s1, Figure S1: FLD chromatograms of FMOC-α-AA standard compound (A) and of a real sample in which FMOC-α-AA is manually integrated (B).
