# Supplementary material for: Allysine and α-Aminoadipic Acid as Markers of the Glyco-Oxidative Damage to Human Serum Albumin under Pathological Glucose Concentrations
Source: Antioxidants (Basel). 2021 Mar 17;10(3):474. doi: 10.3390/antiox10030474 (PMC8002732; doi:10.3390/antiox10030474)
Supplement: Supplementary file 1 [file antioxidants-10-00474-s001.pdf]

SUPLEMMENTARY MATERIAL

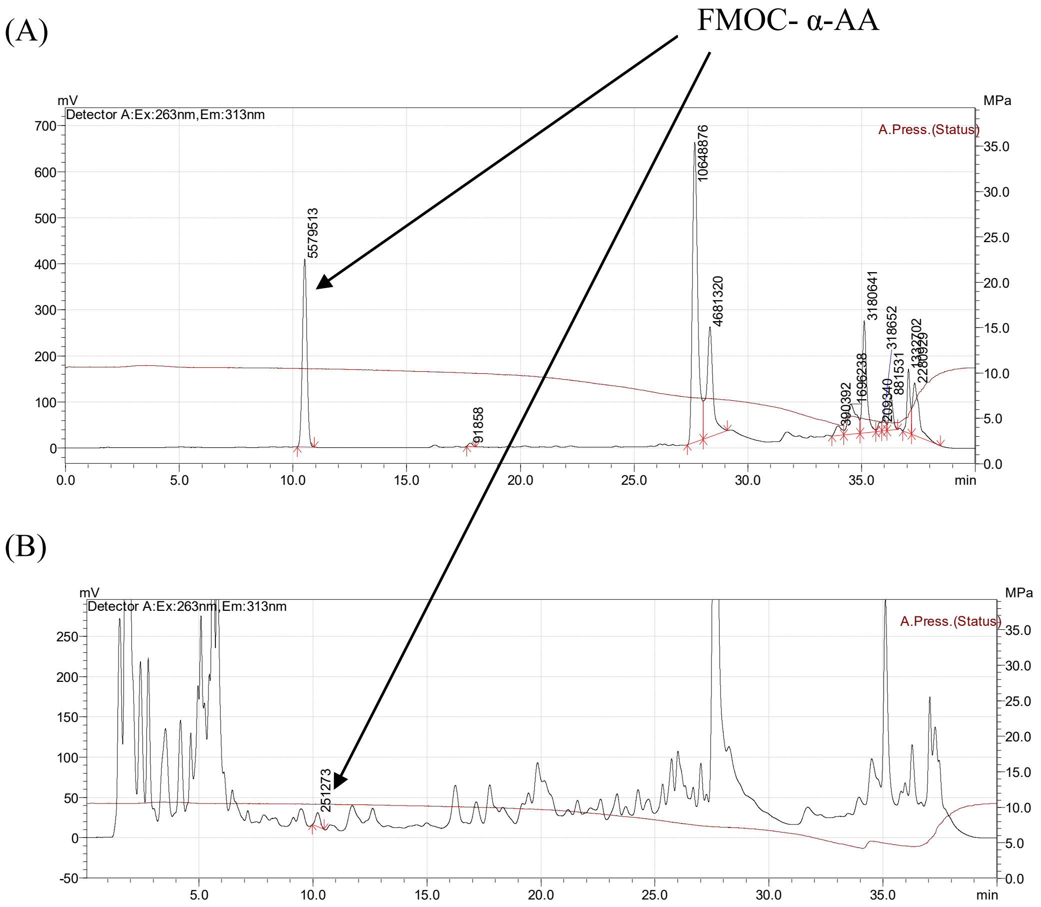

Figure 1. FLD chromatograms of FMOC-α-AA standard compound (A) and a real sample in which FMOC-α-AA is manually integrated (B).
